# Eclampsia and posterior reversible encephalopathy syndrome (PRES): A retrospective review of risk factors and outcomes

**DOI:** 10.5339/qmj.2021.4

**Published:** 2021-02-16

**Authors:** Nissar Shaikh, Shoaib Nawaz, Firdous Ummunisa, Aamir Shahzad, Jazib Hussain, Kiran Ahmad, Haleema S Almohannadi, Hussein Attia Sharara

**Affiliations:** ^1^Department of Anesthesia, SICU, Hamad Medical Corporation, Doha, Qatar; ^2^Department of Anesthesia and Perioperative Medicine, Hamad Medical Corporation, Doha, Qatar E-mail: snawaz1@hamad.qa; ^3^Department of Obstetrics and Gynecology, Women's Wellness and Research Center, Hamad Medical Corporation, Doha, Qatar; ^4^Department of Internal Medicine, Hamad Medical Corporation, Doha, Qatar; ^5^Department of Communicable Diseases, Hamad Medical Corporation, Doha, Qatar; ^6^Department of Obstetrics and Gynecology, AlKhor Hospital, Hamad Medical Corporation, Qatar

**Keywords:** eclampsia, antenatal, eclampsia, cesarean section, gestational age, multiparous, post-partum, posterior reversible encephalopathy syndrome

## Abstract

Posterior reversible encephalopathy syndrome (PRES) is a clinical and radiological entity initially described in 1996. PRES frequently develops in patients with preeclampsia and eclampsia. There is not much literature on risk factors causing PRES in pregnant patients with eclampsia. This study aimed to determine the incidence of PRES in eclampsia, its association with pregnancy, risk factors, and maternal and perinatal outcomes.

Patients and methods: All patients who were admitted with eclampsia and developed PRES in an intensive care unit of a tertiary medical facility between 1997 and 2017 were included in the study. Patients’ demographics, pregnancy and gestational data, treatment mode, and outcomes were retrospectively obtained from their medical charts/files. Data were entered using SPSS program version 23. Chi-square test was used to compare the variables, and a *p* value of < 0.05 was considered statistically significant.

Results: A total of 151 patients were admitted during the study period, and 25 developed PRES. The diagnosis was common in patients older than 25 years. Eclampsia patients who developed PRES were without any pregnancy-associated comorbidities (*p* < 0.08). At the time of diagnosis, their gestational age was more than 36 weeks, which was significant (*p* < 0.04). Incidence was significantly higher in patients presenting with eclampsia and had recurrent seizures (*p* < 0.01 and 0.002, respectively). Its incidence was significantly higher in postpartum eclampsia patients (*p* < 0.01). It was also significantly higher in patients who had cesarean section and hypertension treated with labetalol (*p* < 0.001 and 0.02, respectively). Overall, the maternal mortality rate of eclampsia patients complicated with PRES was 4% in our population.

Conclusion: Of eclampsia patients, 16% developed PRES, which is on the lower side than the reviewed literature (10%–90%). Eclampsia on presentation, recurrent seizures, postpartum eclampsia, cesarean delivery, and labetalol use were associated with increased risk of PRES development.

## Introduction

Posterior reversible encephalopathy syndrome (PRES) commonly manifests as a confusional state, convulsion, or acute blindness.^1,2^

Magnetic resonance imaging (MRI) usually shows typical bilateral white matter changes. Usually, these clinical and radiological changes are reversible in two to three weeks.^1,3^ PRES is commonly caused by severe hypertension, eclampsia, preeclampsia, sepsis, renal failure, and immunosuppressive therapy.^3,4^ Eclampsia is an important and frequent etiology of PRES.^5,6^ There is a scarcity of literature about pregnancy- and eclampsia-related risk factors for developing PRES in eclampsia patients.^3,4,7^ Brewer et al.,^4^ found that 46 of 47 eclampsia patients had PRES syndrome; however, they did not find any significant differences in the incidence between ethnicity groups, maternal age, or gestational age.

This study aimed to determine the incidence of PRES in eclampsia, its association with pregnancy, risk factors, and maternal and perinatal outcomes.

## Patients And Methods

After obtaining permission for the eclampsia study from the department and the institutional review board (IRB Research proposal Number: 10044/10), all eclampsia patients diagnosed with PRES, from 1997 to 2017, were included in the study. The data were gathered as a chart review, which was conducted retrospectively. All patients who were admitted to the hospital with the diagnosis of eclampsia within the study period were included in the review. The project research team, consisting of five members, reviewed all charts with eclampsia as their primary diagnosis. These charts were randomly selected from all eclampsia patients admitted to the hospital. Of 151 patients who developed eclampsia, only 25 were diagnosed with PRES, and these patients were used in the statistical analysis. These patients were included in the study according to the definitions of the diseases in the Definitions section. All patients were managed in an intensive care unit (ICU) and underwent MRI for the diagnosis of PRES. Computed tomography (CT) scan was also performed for these patients, and an abnormal CT scan included brain infarction in the posterior, parietal, and occipital areas. Patient's demographic data, comorbidities, gestational weeks, parity, fetal delivery mode, imaging studies, antihypertensive and anticonvulsant medications, and maternal and perinatal outcomes were recorded retrospectively.

### Inclusion criteria

All pregnant patients who were diagnosed with eclampsia and developed PRES were included in the study.

### Exclusion criteria

Patients who had a diagnosis of PRES due to any other primary conditions such as sepsis, hypertension, and immunosuppression therapy were excluded from the study.

### Definitions

PRES in eclampsia patients is diagnosed with clinical features of altered mental status, headache, and/or visual disturbances along with MRI, findings of bilateral symmetrical hyperintensities on tbl2-weighted images in the parietal and occipital lobes, and no other alternative differential diagnosis consistent with PRES.^8^

Glasgow Coma Scale (GCS) is a neurological scale that aims to provide a reliable and objective way of recording a person's state of consciousness for initial and subsequent assessment. It is used to assess PRES as most patients who develop this syndrome present with a drop in their GCS scores.

Number of fits were categorized into only single episode (1), two episodes, or more than two (multiple) episodes of seizures.

Antenatal visits were divided into two as follows: less than 10 visits were categorized as not regularly followed and 10 or more visits as regular antenatal care. This was taken from the guidelines of the World Health Organization regarding antenatal visits.

### Hypertensive disorders of pregnancy

These include all of the following mentioned entities: preeclampsia, all eclampsia types, hemolysis, elevated liver enzymes, and low platelets (HELLP) syndrome, and gestational hypertension. Further definitions are given as follows:

Preeclampsia refers to the new onset of hypertension and proteinuria or the new onset of hypertension and significant end-organ dysfunction with or without proteinuria after 20 weeks’ gestation in a previously normotensive woman. Hypertension is defined in pregnancy as systolic blood pressure ≥ 140 mmHg and/or diastolic blood pressure ≥ 90 mmHg.

Eclampsia refers to the occurrence of a grand mal seizure in a woman with preeclampsia in the absence of other neurological conditions that could account for seizure.

Antepartum eclampsia is defined as having eclampsia from 20 weeks’ gestation to labor onset. Intrapartum eclampsia is defined as the occurrence of seizures during normal delivery or lower segment cesarean section. Postpartum eclampsia is defined as the occurrence of seizures within 10 days of delivery.

HELPP syndrome is a complication of late pregnancy characterized by hemolysis, elevated liver enzymes, and low platelets.

Chronic hypertension in pregnancy is diagnosed before 20 weeks’ gestation and gestational hypertension after 20 weeks’ gestation.

All these definitions are important to understand since the primary diagnosis of eclampsia and PRES is based on these definitions. All patients who were positively diagnosed with PRES and eclampsia according to the above definitions were included in this study.

### Statistical analysis

Data were entered and analyzed using IBM SPSS version 23. Descriptive statistics in the form of mean and standard deviation were performed for interval variables. Frequency with percentages was calculated for categorical variables. Chi-square tests were performed to determine the association between categorical variables. Student *t*-tests (unpaired) were performed to determine statistically significant mean differences between interval variables. The interval variables were detected as normal using the Kolmogorov–Smirnov tests. Multivariate analysis could not be performed because of inappropriate sample size. A *p* value of ≤ 0.05 (two tailed) was considered statistically significant.

### Results

**Figure 1.**
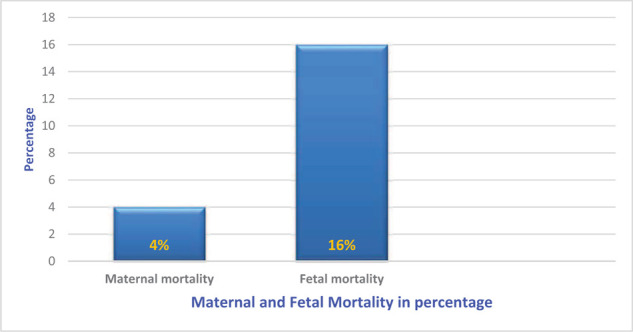
The percentage of maternal and fetal mortality in posterior reversible encephalopathy syndrome.

The total number of deliveries during the study period was 463,016, from which 151 patients had eclampsia and 25 PRES. The average GCS score was 14 ± 1. Of patients, 60% were more than 25 years old (Figure 1).

**Figure 2.**
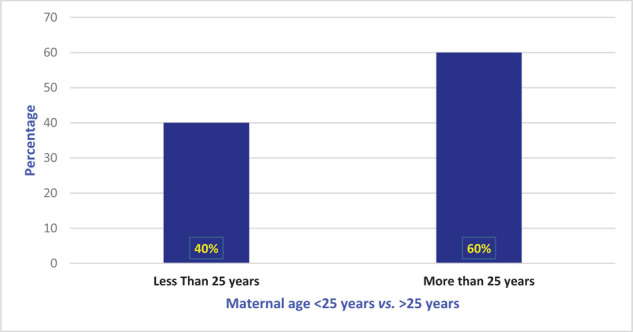
Posterior reversible encephalopathy syndrome and patients’ age.

PRES eclampsia was less common in the local population (40% *vs.* 60%), and it was significantly higher (*p* < 0.008) in patients with no pregnancy-associated comorbidity. PRES was more common in multiparous patients (52% *vs.* 48%). The incidence was significantly higher (*p* < 0.04) after 36 weeks’ gestation. It was significantly higher despite regular antenatal care (*p* < 0.002). The incidence was significantly higher in patients with eclampsia than in those with preeclampsia (*p* < 0.01). The common mode of fetal delivery was lower segment cesarean section, and these patients had a significantly higher incidence of PRES (*p* < 0.001). PRES was significantly higher in postpartum eclampsia patients (*p* < 0.01) than in prepartum and intrapartum eclampsia patients. The incidence was significantly higher (*p* < 0.02) in patients with recurrent seizures (Table 1). Of patients, 20% received magnesium sulfate (MgSO_4_), 44% benzodiazepines in addition to MgSO_4_, 16% MgSO_4_ and an anticonvulsant, and 20% MgSO_4_, benzodiazepines, and anticonvulsants to control seizures (Table 1). PRES was significantly higher when labetalol was used to control hypertension in eclampsia patients (*p* < 0.02; Table1). CT brain scans of PRES eclampsia patients were normal in 44% and showed infarction in the posterior, parietal, or occipital areas in 56%. In all patients, MRI was diagnostic of PRES (Table 1). Most PRES patients were discharged with antihypertensive and anticonvulsant medications (Table 1). The average length of ICU stay of PRES eclampsia patients was 4 ± 2 days. The maternal mortality rate was 4% as 1 of 25 patients died with HELLP syndrome and severe thrombocytopenia complications. The perinatal mortality rate was 16% (4 of 25 fetal deaths; Figure 2). These rates were calculated considering our sample size of 25 patients with diagnosis of PRES.

## Discussion

PRES is a potentially reversible neurological entity, presenting with altered consciousness, acute cortical blindness, and convulsions. Delayed diagnosis can lead to poor prognostic indicators.^9^ Typical imaging study findings include vasogenic edema with or without ischemic changes in the posterior brain circulation.^10^

There is limited knowledge regarding the development of PRES in eclampsia patients and its associated risk factors from our region.^4,5,11–13^

Eclampsia is a common etiological reason for developing PRES.^6^ It is well described in the literature that these patients with hypertensive disorders of pregnancy are at a higher risk of developing PRES as they have episodes of hypertension and etiopathology of vascular nature, in conjunction with disturbed blood brain barrier and vasogenic brain edema.^4–6^ This study aimed to assess pregnancy-related risk factors and their association with the development of PRES. This study aimed to determine the associated risk factors, address them in the antenatal period, and improve outcomes by preventing PRES in these high-risk groups.

The rate of eclampsia patients who developed PRES was lower in this study (16%) than in Wen et al.’s^14^ study (92.85%).^4^ In contrast, Bembalgi et al.,^15^ reported a significantly lesser incidence of PRES in eclampsia patients (0.03%), although they did not mention whether these patients were managed in an intensive care setup or ward. The maternal age of eclampsia patients developing PRES in this study was significantly higher than in the literature. Bembalgi et al.,^12^ had younger eclampsia patients developing PRES (20–25 years old). Fisher et al.,^15^ also described younger maternal age as a risk for developing PRES in eclampsia patients. Although various risk factors for developing PRES in eclampsia patients are described in this study, 56% of PRES eclampsia patients had no comorbidities and pregnancy-associated risk factors, 28% had preeclampsia, and 16% had gestational diabetes mellitus. Roth et al.,^16^ also compared PRES patients with and without pregnancy and reported that 75% of the pregnant patients were without any comorbidity, one patient had hypertension, and one had obesity, whereas 23% of nonpregnant patients developing PRES had diabetes mellitus. Most of the PRES eclampsia patients in this study were multiparous. In the literature, PRES is described to be common in primiparous patients.^12,17^ Postpartum eclampsia patients had a significantly higher incidence of PRES in this study than in Mavani et al.’s^17^ study, although Bembalgi et al.,^15^ also reported a higher incidence.

Most patients had single or recurrent episodes of seizure activity in contrast to what was described in the literature.^4,12,17^ It may be related to the use of MgSO_4_ in this study as it reduces the occurrence of recurrent seizures. The eclampsia patients who developed PRES in this study had a significantly higher recurrent seizure activity. The common mode of fetal delivery was lower segment cesarean section, which was also described in the literature.^15^ The gold standard for the diagnosis of PRES is MRI; in this study, 44% of the PRES patients had normal CT brain, but all had PRES confirmation by MRI.^18^ Initially, CT scan was performed in these patients to rule out gross central nervous system (CNS) pathology such as hemorrhage or infarction. Subsequently, these patients underwent MRI for final diagnosis.

In this study, eclampsia patients treated with labetalol infusion to control blood pressure had a significantly higher rate of PRES, and this may be related to the effects of β-blockers on the CNS. A prospective randomized study will be needed to confirm this finding.

PRES in general has a good outcome. One PRES eclampsia mother died in this study; she had HELLP syndrome apart from PRES and eclampsia with severe thrombocytopenia. Wen et al.,^14^ also reported a maternal mortality of 7.69%, and both patients had disseminated intravascular coagulopathy. Bambalgi et al.,^12^ had a better maternal outcome for PRES eclampsia patients but had a higher perinatal mortality (27.3%), whereas Fugate et al.,^19^ reported a mortality rate of up to 6%. In our study, the perinatal mortality was 16%.

## Limitations

Limitations of this study include that it is a single-center retrospective study and has a comparatively small sample size. Further prospective multicenter studies are required for better quality data and recommendation.

Binary regression analysis to assess the risk factors associated with the diagnosis could not be conducted because of the small sample size.

PRES incidence did not decrease despite regular antenatal care in our patients. Another prospective large multicenter study is needed to determine the possible reasons.

## Conclusions

In this study, eclampsia patients who developed PRES had higher gestational age and recurrent seizure episodes. They also had a higher number of cesarean deliveries, postpartum eclampsia, and labetalol use to control blood pressure. The absence of pregnancy-associated comorbidities did not decrease the incidence of PRES in eclampsia patients.

In the end, a low threshold is recommended for the possibility of diagnosing PRES in eclampsia patients who develop neurological symptoms, particularly in patients having the abovementioned risk factors. This neurological disorder is potentially reversible with early diagnosis and appropriate management.

## Figures and Tables

**Table 1:** Posterior reversible encephalopathy syndrome (PRES) and variables.

	Patients (n=25)	Percentage (%)	p value

PRES and nationality			

Local	10	40	0.15

Expatriate	15	60	

PRES and pregnancy-associated diseases			

None	14	56	

Preeclampsia	7	28	0.008

Gestational diabetes mellitus	4	16	

PRES and parity			

Primiparous	12	48	0

Multiparous	13	52	0.8

PRES and gestational age			

Less than 36 weeks	9	36	

More than 36 weeks	16	64	0.04

PRES and antenatal visits			

None	4	16	

Less than 10	6	24	0.002

More than 10	15	60	

PRES and eclampsia type			

Antepartum	10	40	

Intrapartum	3	12	0.01

Postpartum	12	48	

PRES and admission diagnosis			

Preeclampsia era	8	32	

Eclampsia	17	68	0.01

PRES and number of fits			

Once	10	40	

Twice	3	12	0.02

Recurrent	12	44	

PRES and mode of deliveries			

Lower section cesarean section	19	76	

Normal vaginal	5	20	0.001

Assisted vaginal	1	4	

PRES and anticonvulsants			

Magnesium sulfate	5	20	

Magnesium sulfate+benzodiazepines	11	44	0.08

Magnesium sulfate+anticonvulsants	4	16	

Magnesium sulfate+benzodiazepines+anticonvulsants	5	20	0.08

PRES and antihypertensive			

Labetalol	13	52	

Hydralazine	4	16	

Labetalol+hydralazine	8	32	0.02

PRES and medication on discharge			

None	5	20	

Labetalol	10	40	0.22

Keppra (leviteracetam)	10	40	

PRES and computed tomography findings			

Normal	11	44	

Abnormal	14	66	0.3

